# Right ventricular diastolic adaptation to pressure overload in different rat strains

**DOI:** 10.14814/phy2.16132

**Published:** 2024-07-11

**Authors:** Julie S. Axelsen, Stine Andersen, Steffen Ringgaard, Rowan Smal, Aida Lluciá‐Valldeperas, Jens Erik Nielsen‐Kudsk, Frances S. de Man, Asger Andersen

**Affiliations:** ^1^ Department of Cardiology Aarhus University Hospital Aarhus Denmark; ^2^ Department of Clinical Medicine Aarhus University Aarhus Denmark; ^3^ MR Research Center Aarhus University Aarhus Denmark; ^4^ Department of Pulmonary Medicine PHEniX Laboratory, Amsterdam UMC, Locatie VUmc Amsterdam The Netherlands

**Keywords:** diastolic dysfunction, pulmonary trunk banding, rat strains, right ventricular failure

## Abstract

Different rat strains are used in various animal models of pulmonary hypertension and right ventricular (RV) failure. No systematic assessment has been made to test differences in RV response to pressure overload between rat strains. We compared RV adaptation to pulmonary trunk banding (PTB) in Wistar (W), Sprague Dawley (SD), and Fischer344 (F) rats by hemodynamic profiling focusing on diastolic function. Age‐matched male rat weanlings were randomized to sham surgery (W‐sham, *n* = 5; SD‐sham, *n* = 4; F‐sham, *n* = 4) or PTB (W‐PTB, *n* = 8; SD‐PTB, *n* = 8; F‐PTB, *n* = 8). RV function was evaluated after 5 weeks by echocardiography, cardiac MRI, and invasive pressure‐volume measurements. PTB caused RV failure and increased RV systolic pressures four‐fold in all three PTB groups compared with sham. W‐ and SD‐PTB had a 2.4‐fold increase in RV end‐systolic volume index compared with sham, while F‐PTB rats were less affected. Diastolic and right atrial impairment were evident by increased RV end‐diastolic elastance, filling pressure, and E/e' in PTB rats compared with sham, again F‐PTB the least affected. In conclusions, PTB caused RV failure with signs of diastolic dysfunction. Despite a similar increase in RV systolic pressure, F‐PTB rats showed less RV dilatation and a more preserved diastolic function compared with W‐ and SD‐PTB.

## 1 INTRODUCTION

Right ventricular (RV) failure is the prime cause of death in patients with pulmonary hypertension (PH) (Borgdorff et al., 2015; Haddad et al., 2008). Robust and well‐characterized animal models of PH and RV failure are essential in order to investigate new treatment strategies and underlying disease mechanisms. The three most used animal models are the monocrotaline (MCT), the sugen‐hypoxia (SuHx), and the pulmonary trunk banding (PTB) models. Both the MCT and SuHx models primarily mimic PH and secondarily RV failure. On the other hand, PTB is a model of isolated RV failure after applying a clip or ligature around the pulmonary trunk (PT). Each model has its strengths and limitations, so to improve the applicability of a study it is often essential to include several models.

The use of different rat strains in these models hampers direct comparisons, as the strains' different genetic backgrounds may affect how well the RV adapts to pressure overload (Bochnowicz et al., 2000; Ramot et al., 2015; Ward & Kodavanti, 2015). We already know that rat strain differences exist and may influence study results (Depuydt et al., 1999; Ebmeyer et al., 2002). Wistar and Sprague Dawley rats are the two most frequently used rat strains in animal models of PH and RV failure (Dignam et al., 2022). On the other hand, Fisher344 rats have shown highly increased mortality when utilized in the SuHx model compared with Sprague Dawley rats (Jiang et al., 2016; Lee et al., 2020; Shults et al., 2019). Until now, no systematic assessment has been performed to test differences in RV response to pressure overload between these rat strains.

In recent years, the importance and clinical impact of RV diastolic dysfunction in RV failure has been established, but its mechanisms and precise role in RV failure development remain poorly described (Axell et al., 2015). Studies of the left ventricle (LV) show that LV diastolic dysfunction is essential in disease development. Unfortunately, this situation is not directly transferable to the RV due to the different geometry and pressure system (Jung et al., 2021). Only a few studies have assessed and documented RV diastolic stiffness and dysfunction in patients with PH (Inampudi et al., 2020; Jung et al., 2021; Rain et al., 2013; Vanderpool et al., 2022; Wessels et al., 2021). Diastolic stiffening is a prognostic indicator of diastolic dysfunction and may precede RV systolic dysfunction in patients with PH (Jung et al., 2021). Further, RV diastolic dysfunction may predict disease progression and disease severity (Rain et al., 2013; Shiina et al., 2009; Vanderpool et al., 2022). RV stiffness has shown to be an important contributor to RV failure in several animal models of PH and RV failure (Alaa et al., 2016; Gaynor et al., 2005; Kwan et al., 2021).

In the present study, we compared RV adaptation to PTB in Wistar, Sprague Dawley, and Fischer344 rats by hemodynamic profiling and characterization of RV tissue remodeling focusing on diastolic function.

## 2 MATERIALS AND METHODS

### 2.1 Animals

Male Wistar, Sprague Dawley, and Fischer344 rats (Janvier Labs, Hannover) had ad libitum access to standard rat chow (Altromin #1324; Altromin, Lage, Germany) and drinking water. The rats were housed two per cage (open‐top, type III cage) kept in a cabinet (Scantainer) in a room with 21°C and a 12‐hour light–dark cycle. Each cage had bedding material (Abedd, Espe Mini 1–2 mm), environmental enrichment (shelter, gnawing brick, snacks), and nesting materials (sizzle nest, paper wool, and cocoon (Datesand)). The rats were treated according to Danish national guidelines, and all experiments were approved by the Institutional Ethics Review Board, the Danish Animal Experiments Inspectorate, and conducted in accordance with the Danish law for animal research (authorization number 2021‐15‐0201‐00928, Ministry of Environment and Food of Denmark). The veterinary staff monitored the rats daily, and health was monitored by general assessment of animal activity, respiration, and fur condition.

### 2.2 Study design

**FIGURE 1:**
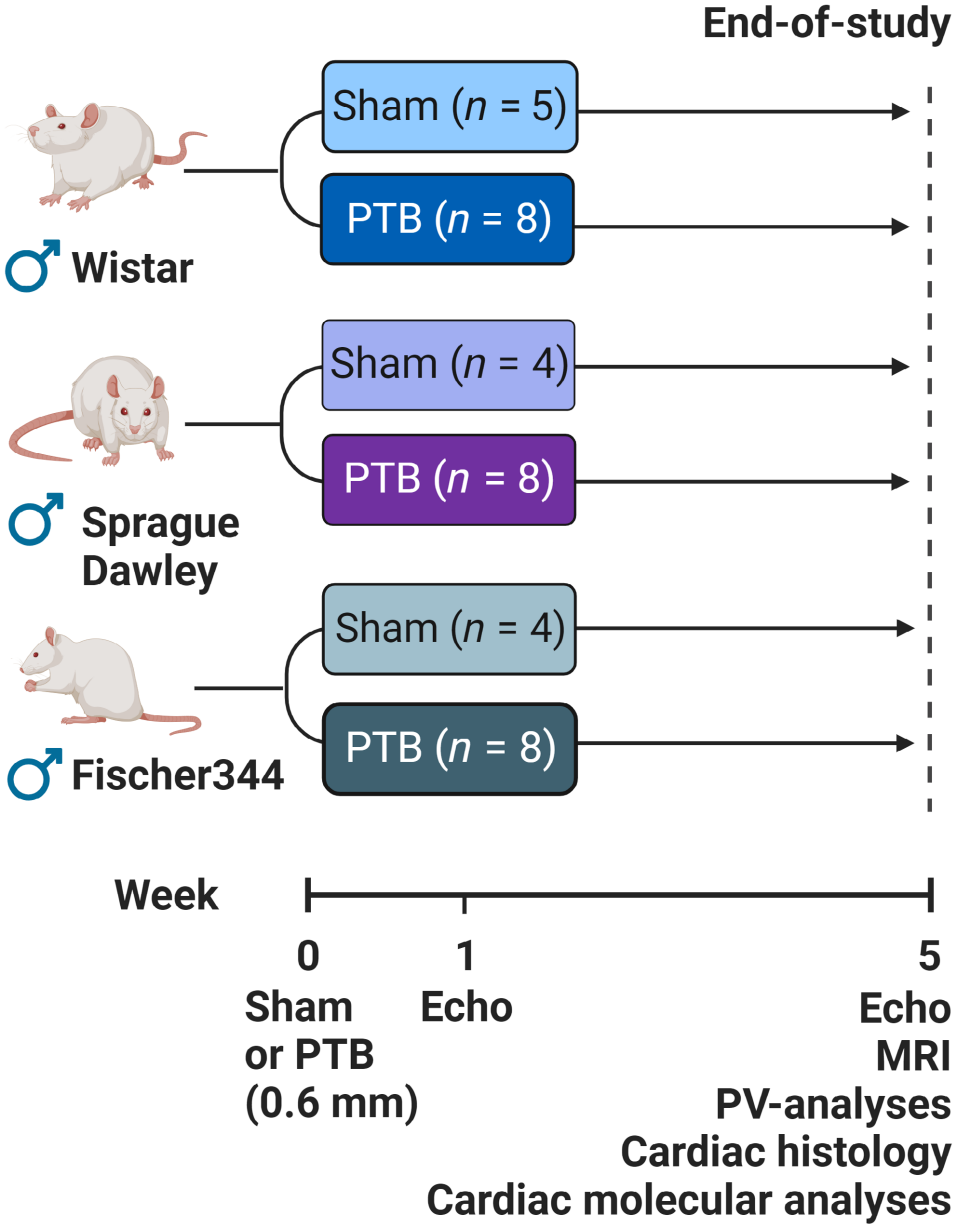
Study design. Male Wistar, Sprague Dawley, and Fischer344 rats were randomized to either sham or pulmonary trunk banding (PTB) surgery. One week after the surgery, an echocardiography (echo) was performed on all the rats. Five weeks after surgery, cardiac function was evaluated by echocardiography, magnetic resonance imaging (MRI), and invasive pressure‐volume (PV) measurements. Afterwards the rats were euthanized, and histochemical and molecular analyses were performed on the hearts. Figure created with BioRender.com.

After one week of acclimatization, RV failure was induced by PTB surgery. Approximately 4.5 weeks old male Wistar, Sprague Dawley, and Fischer344 rats were randomized to either PTB or sham surgery with the operator blinded for rat strain. A total of six groups were included: Wistar sham (W‐sham, *n* = 6); Wistar PTB (W‐PTB, *n* = 9); Sprague Dawley sham (SD‐sham, *n* = 4); Sprague Dawley PTB (SD‐PTB, *n* = 8); Fischer344 sham (F‐sham, *n* = 4); and Fischer344 PTB (F‐PTB, *n* = 8). One week after surgery, an echocardiography was performed in all rats to verify the development of RV dysfunction in PTB rats. Five weeks after surgery, RV systolic and diastolic function were evaluated by hemodynamic profiling consisting of echocardiography, magnetic resonance imaging (MRI), and invasive pressure‐volume measurements. Afterwards, the rats were euthanized by exsanguination, the hearts excised, and histochemical and molecular analyses were performed to assess RV remodeling (Figure 1). The order of the rats was randomized on each day of the experiments. All procedures were performed by one operator (JSA) and analyzes were done by one trained reader (JSA) blinded to the source of the images (Gouma et al., 2012). All relevant measures have been size‐corrected with body surface area (BSA), BSA=body weight23/100.

### 2.3 Pulmonary trunk banding

Banding of the PT was performed as described previously (Andersen et al., 2018). Briefly, the rat weanlings were anesthetized with sevoflurane (Sevorane, AbbVIE Inc; 7% induction, 3.5% maintenance in a 2:1 O_2_/air mix). After induction of anesthesia, the rats were intubated and connected to a mechanical ventilator (Ugo Basile 7025, rodent ventilator; respiration frequency of 76 per minute and tidal volume 1.5 mL). The rats were injected with analgesics (s.c. buprenorphine 0.1 mg/kg, Temgesic, Indivior Europe limited, Ireland and s.c. carprofen 5 mg/kg, ScanVet Animal Health, Fredensborg, Denmark). After a lateral thoracotomy, the PT was carefully separated from the ascending aorta, and the banding was made with a ligating clip applier modified to compress the titanium clip to a preset inner diameter of 0.6 mm. The thorax was closed in three layers and the rats injected with 2 mL isotonic saline solution s.c. to compensate for fluid loss. To relieve postoperative pain, the rats were treated with buprenorphine in the drinking water (7.4 μg/mL) for 3 days. Except for the application of the clip, the sham rats underwent the same surgery.

### 2.4 Echocardiography

A transthoracic echocardiography was performed 1 and 5 weeks after surgery. The rats were sedated with sevoflurane (Sevorane, AbbVIE Inc; 7% induction, 3.0–3.5% maintenance). The scans were performed on a Vevo 2100 Imaging System (VisualSonics Inc, Toronto, ON, Canada) scanning at 14–21 MHz using a MS250 line array transducer. In the parasternal long axis view, pulsed wave Doppler was used to measure velocity time integral (VTI, an average of three systematically chosen points) in the PT. Stroke volume (SV) was calculated as: $\mathrm{SV}={\left(\frac{\mathrm{PT}\;\text{diameter}}{2}\right)}^{2}\cdot \pi \cdot \mathrm{VTI}$. In an apical 4‐chamber view, following measures were obtained: Tricuspid annular plane systolic excursion (TAPSE); RV inflow velocities using pulse wave doppler at the tip of the tricuspid leaflets to assess diastolic function; tissue doppler imaging of the lateral tricuspid annulus; And right atrial area indexed to BSA (RAAi). To minimize beat‐to‐beat variation all parameters were measured in three consecutive heart cycles. All images were analyzed off‐line (Vevo® 2100 software v. 5.6.0, Fujifilm VisualSonics Inc, Amsterdam, The Netherlands).

### 2.5 Magnetic resonance imaging

A 9.4 Tesla Agilent MRI system with a volume transmit/receive rat coil was used to measure RV volume, RV ejection fraction (calculated as the percentage change in end‐diastolic and end‐systolic volume), and cardiac index (CI, calculated as CI = cardiac output/BSA). The imaging was synchronized to heartbeat and respiration using a trigger system (SAII, New York, USA). For volume measurements, a set of short axis cine images was obtained covering the entire RV and right atrium (RA) (slice thickness of 1.5 mm and in‐plane resolution of 0.2 mm). The RV endocardium was drawn manually on each slice during a heart cycle and hereby obtaining values of end‐systolic (ESV) and end‐diastolic volumes (EDV) using Segment v3.3 R9405e (http://segment.heiberg.se) (Heiberg et al., 2010). Global circumferential and longitudinal RV strains were calculated offline by feature tracking using the strain analyses module in Segment. For global circumferential strain, endocardial and epicardial borders were manually drawn in short‐axis views of the basal, mid, and apical RV at end‐diastole. Same was applied for global longitudinal strain in 4‐chamber, RV long axis, and RV 3‐chamber views. The software then automatically identified endocardial borders throughout the heart cycle and computed mean global myocardial strain as an average of the strain curves in the beforementioned views. A phase contrast flow measurement was obtained (slice thickness of 2.0 mm and in‐plane resolution of 0.2 mm) and blood flow in the pulmonary artery was calculated with the specially made analysis software Siswin.

### 2.6 Invasive pressure‐volume measurements

The rats were anesthetized, intubated, and ventilated (respiration frequency of 76 per minute and tidal volume of approximately 3.7 mL). To prevent blood clotting during the procedure, the rats were injected with 50 units of heparin i.m. (Heparin, Leo Pharma A/S, Ballerup, Denmark). Systemic blood pressures were measured after stabilization by a catheter inserted in the right carotid artery. RV pressure‐volume loops were obtained by a conductance catheter (SPR‐869, Millar Instruments, Houston, TX) and processed in Powerlab 16/35 (AD Instruments, UK, sampling rate of 2 kHz (Ogilvie et al., 2020)) using the open chest approach. Steady state RV pressures were recorded after stabilization. By slowly occluding the inferior vena cava simultaneous recordings of RV pressures and volumes with decreasing preloads were obtained producing consecutive pressure‐volume loops. The conductance signal was calibrated using ESV and EDV from MRI volume measurements. Labchart software (AD Instruments, UK) was used to calculate load‐independent measures of RV contractility and diastolic function as well as load‐dependent measures.

### 2.7 Euthanasia

The heart was quickly excised, and the atriums and ventricles weighed individually. RV/(LV + S) was used as measure of RV hypertrophy. RV tissue was snap frozen for molecular analyses and immersion fixated in formalin 10% for histology. The thoracic and abdominal cavities were checked for pleural fluid and ascites (>1 g used as cut off). The liver was weighed and examined for dark discoloration (nutmeg liver) as a sign of backward failure. Remaining organs were weighed.

### 2.8 Analyses of cardiac tissue

#### 2.8.1 Histology

Cardiac tissue was embedded in paraffin within 72 hours. For determination of cardiomyocyte cross sectional areas (CSA) 3 μm sections were stained with hematoxylin & eosin (HE) following standard protocol on Ventana HE600 stainer (Roche diagnostics, HE600 hematoxylin 07024282001 and HE600 Eosin 06544304001). The RA and RV cardiomyocyte CSA were calculated in ImageJ (Rasband, WS, ImageJ, US National Institutes of Health, Bethesda, Maryland, USA, https://imagej.nih.gov/ij/, 1997–2018) and expressed as the average CSA of minimum 20 RA or 25 RV cardiomyocytes. The cardiomyocytes were distributed in the section and cut transversally at the level of the nuclei. To visualize fibrosis, another set of sections were stained with collagen specific picrosirius red using an in‐house, routine diagnostic protocol from The Capital Region of Denmark Hospital Pharmacy. RV fibrosis was expressed as the mean percentage tissue area positive for collagen measured over minimum seven randomly chosen areas by adjusting color thresholds in ImageJ. For determination of RV capillary density 5 μm sections were permeabilized, blocked, and counterstained with Hoechst 33342 nuclear dye (diluted 1:500; Santa Cruz Biotechnology), DyLight‐488 conjugated Lycopersicon Esculentum (Tomato) Lectin (diluted 1:200; Thermofisher scientific), and Alexa‐647 conjugated wheat germ agglutinin (WGA, diluted 1:500; Thermo Fisher Scientific). Images were acquired at 20x magnification on the Nikon AXR confocal microscope with the same settings for all animals. Capillary density was quantified on cross‐sectional fibers from minimum four randomly chosen areas of the Lectin stainings through ImageJ. Areas with larger vessels and contaminations were excluded.

#### 2.8.2 Polymerase chain reaction (PCR)

To investigate underlying molecular mechanisms, mRNA expression levels of genes involved in cardiac remodeling were evaluated by real‐time PCR analyses. To lyse the ∼30 mg of snap‐frozen RV tissue, RLT lysis buffer (Qiagen, 79,216) was used in combination with stainless steel beads (Qiagen, 69,989) and TissueLyser II (Qiagen). RNA isolation was performed with the RNeasy mini kit (Qiagen, 74,104) and RNase‐Free DNase set (Qiagen, 79,256) to get pure RNA. The RNA yield was measured with NanoDrop One (ThermoScientific). Total RNA was reverse transcribed into complimentary DNA (cDNA) using iScript cDNA Synthesis Kit (Bio‐rad, 1,708,891) and the Thermal Cycler T100 (Bio‐rad). PCR was performed using iQ SYBR Green Supermix (Bio‐rad, 1,708,886) and C1000 touch Thermal Cyler CFX384 (Bio‐rad) along with specific primers for the following genes: brain natriuretic peptide (BNP); transforming growth factor‐β (TGF‐β); collagen 1, and collagen 3. The results have been converted to normalized expression through the 2^−∆∆Ct^ method (Rao et al., 2013) with the housekeeping genes 18 s and hypoxanthine guanine phoshporibosyltransferase (HPRT) (Table S1).

### 2.9 Statistics

Statistical analyses were performed using GraphPad Prism 9.4.0 for Windows (GraphPad Software, San Diego, California USA, www.graphpad.com). All data were tested for normal distribution by the Shapiro Wilk test. Quantitative data are expressed as mean ± standard deviation (SD) in tables and in scatterplots. In tables, non‐normally distributed data are expressed as median [interquartile range]. Analyses were performed using one‐way ANOVA with Bonferroni post‐hoc comparison or a nonparametric Kruskal Wallis test to evaluate the model (sham vs. PTB) and the differences between the rat strains (W‐sham vs SD‐sham; W‐sham vs F‐sham; SD‐sham vs F‐sham; W‐PTB vs. SD‐PTB; W‐PTB vs. F‐PTB: SD‐PTB vs. F‐PTB). Brown‐Forsythe ANOVA tests were used when significant differences in standard deviation. Dichotomous outcomes were compared between groups by Fisher's exact test. Correlations between end‐diastolic elastance (Eed) and other diastolic measures were performed using the Pearson correlation or the Spearman correlation for nonparametric data. *p* < 0.05 were considered statistically significant.

As the study was explorative the required sample size of 8–9 PTB‐rats were based on experiences from pilot studies and previously published work using the PTB model.

## 3 RESULTS

There was a 20% mortality during PTB surgery, and a total of 40 rats were included in the study. Three animals died prematurely: One F‐PTB rat died 2 weeks after PTB surgery due to acute cardiac failure caused by a wrongly placed clip; one W‐sham died due to anesthetic overexposure during echocardiography at week five; and one W‐PTB died during MRI scanning due to heart failure with excessive pleural fluid. There were no significant differences in mortality between the groups.

### 3.1 Right ventricular systolic function

As expected, the Fischer344 rats weighed 25% less than the two other rat strains at end‐of‐study due to their slower growth curve (Table 1). A clip with an inner diameter of 0.6 mm was applied in all PTB rats, and no differences in pulmonary diameters were observed between rat strains at 1 week or at end‐of‐study indicating an equal strain on the heart (Table S2 and S3).

**TABLE 1:** Anatomic and hemodynamic data at end‐of‐study (5 weeks after PTB surgery).

	W‐sham *n* = 5	W‐PTB *n* = 8	SD‐sham *n* = 4	SD‐PTB *n* = 8	F‐sham *n* = 4	F‐PTB *n* = 8
Anatomical data						
BW at surgery (g)	130 [129–137]	135 [111–140]^i^	142 [127–149]	127 [119–129]	112 [110–113]	105 [98–110]
BW at end‐of‐study (g)	373 ± 41^f^	339 ± 40^i^	411 ± 34^g^	369 ± 36^j^	277 ± 4	273 ± 26
(LV + S)/BSA (g/m^2^)	1.56 ± 0.20	1.63 ± 0.14^h^	1.46 ± 0.09	1.52 ± 0.14	1.33 ± 0.01	1.42 ± 0.07
RA CSA (μm^2^)	236 ± 43	451 ± 48^d^	262 ± 15	446 ± 68^c^	291 ± 71	410 ± 70^a^
EC manifestations	0 (0)	5 (56)^a^	0 (0)	6 (75)	0 (0)	2 (25)
TR	0 (0)	5 (56)^a^	0 (0)	7 (88)^a^	0 (0)	3 (38)
Hemodynamic measures						
RV systolic pressure (mmHg)	23 ± 2.3	84 ± 9.4^d^	22 ± 2.4	102 ± 28.0^c^	25 ± 1.9	85 ± 17.6^c^
RV‐EF (%)	77 [68–78]	45 [39–55]^a^	76 [73–81]	51 [45–58]	82 [77–86]	68 [54–77]
Tricuspid S′ (mm/s)	67 ± 10	41 ± 7^d^	72 ± 6	40 ± 7^d^	70 ± 6	44 ± 6^d^
RV dP/dt max (mmHg/s)	1153 ± 268	3394 ± 614^b^	1146 ± 209	4649 ± 1867^d^	1627 ± 261	3868 ± 1039^a^
HR (bpm)	390 ± 32	350 ± 48	389 ± 7	337 ± 26	428 ± 21	374 ± 37
RV SVI (mL/m^2^)	0.77 [0.73–0.82]	0.57 [0.48–0.68]	0.74 [0.66–0.82]	0.58 [0.55–0.61]	0.66 [0.59–0.81]	0.60 [0.55–0.61]
RA SVI (μL/m^2^)	0.25 ± 0.08	0.24 ± 0.08	0.15 ± 0.02	0.20 ± 0.08	0.22 ± 0.12	0.24 ± 0.06
MAP (mmHg)	117 ± 8^e^	123 ± 15	132 ± 5	122 ± 11	151 ± 18	133 ± 16

Abbreviations: BSA, body surface area; BW, body weight; CSA, cross sectional area; dP/dt max, first derivative (maximal) of right ventricular systolic pressure; EC, Extracardiac; EC manifestations, nutmeg liver, ascites, and/or hydrothorax; EF, ejection fraction derived from MRI as percentage change in end‐diastolic and end‐systolic volume; F, Fischer344; HR, heart rate; LV + S, left ventricle + septum; MAP, Mean arterial pressure; RA SVI (data missing from one W‐sham, one F‐sham, and one F‐PTB due to low scanning quality); PTB, pulmonary trunk banding; RA, right atrium (one missing from W‐sham); RV, right ventricle; SD, Sprague Dawley; SVI, stroke volume index = stroke volume/BSA; TR, tricuspid regurgitation; W, Wistar.

*Note*: Data are presented as mean ± standard deviation or *n* (%). If non‐normally distributed data are presented as median [interquartile range].

^a^ *p* < 0.05.

^b^ *p* < 0.01.

^c^ *p* < 0.001.

^d^ *p* < 0.0001 PTB versus sham within same rat strain.

^e^ *p* < 0.01.

^f^ *p* < 0.001.

^g^ *p* < 0.0001 sham versus F‐sham.

^h^ *p* < 0.05.

^i^ *p* < 0.01.

^j^ *p* < 0.0001 PTB versus F‐PTB.

**FIGURE 2:**
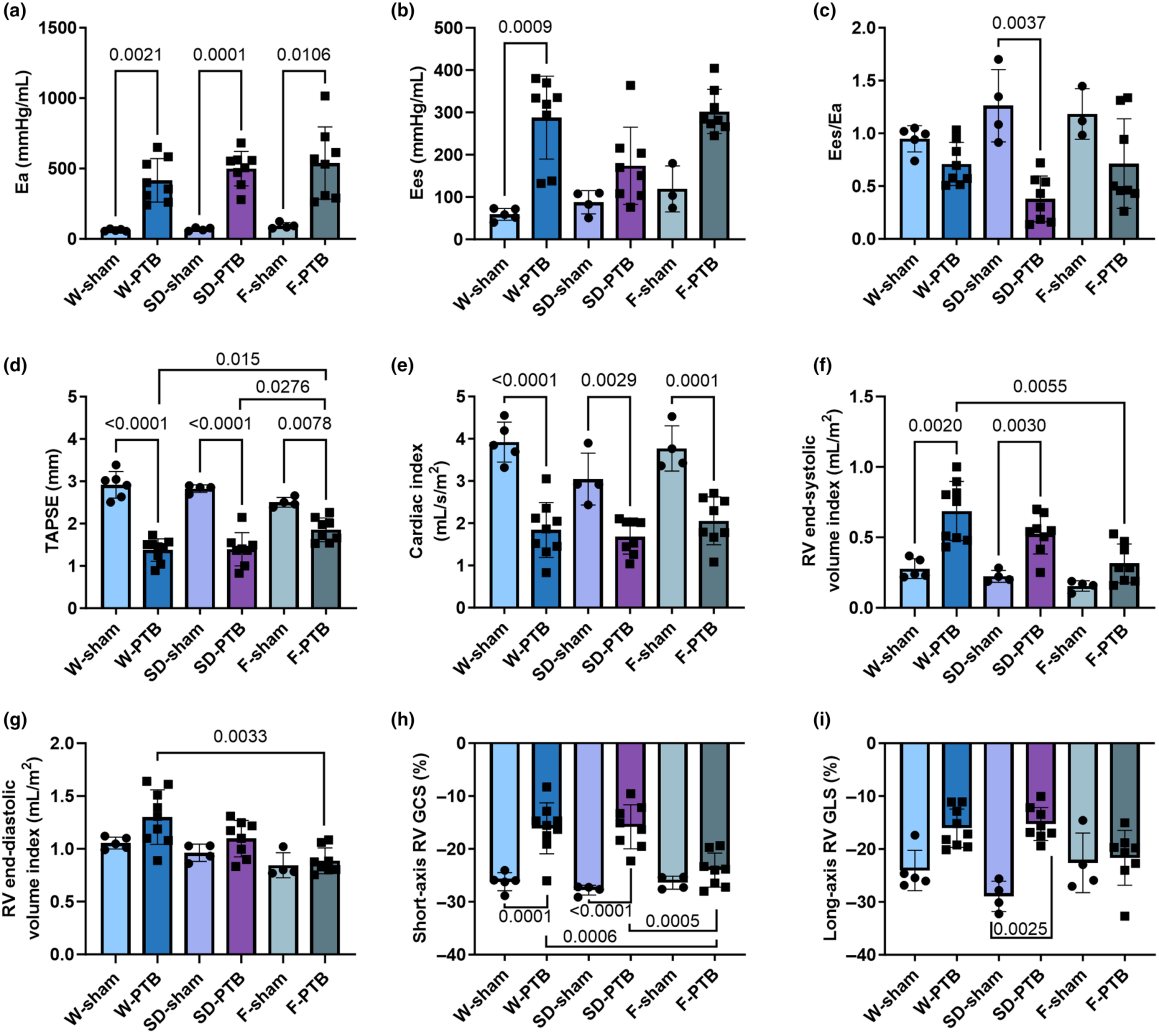
Pulmonary trunk banding caused right ventricular systolic dysfunction at end‐of‐study. W: Wistar; SD: Sprague Dawley; F: Fischer344; PTB: Pulmonary trunk banding. Hemodynamic results from pressure‐volume loops: (a) Arterial elastance; (b) End‐systolic elastance, one outlier excluded in F‐sham; (c) Ventriculo‐arterial coupling, one excluded in F‐sham. (d) Tricuspid annular plane systolic excursion (TAPSE) from echocardiography. Following results derived from MRI: (e) Cardiac index as cardiac output/body surface area (BSA); (f) Right ventricular (RV) end‐systolic volume index (index = volume/BSA); (g) RV end‐diastolic volume index. (h) short‐axis RV global circumferential strain (GCS). (i) Long‐axis RV global longitudinal strain. Results are expressed as scatterplots with mean ± SD.

**FIGURE 3:**
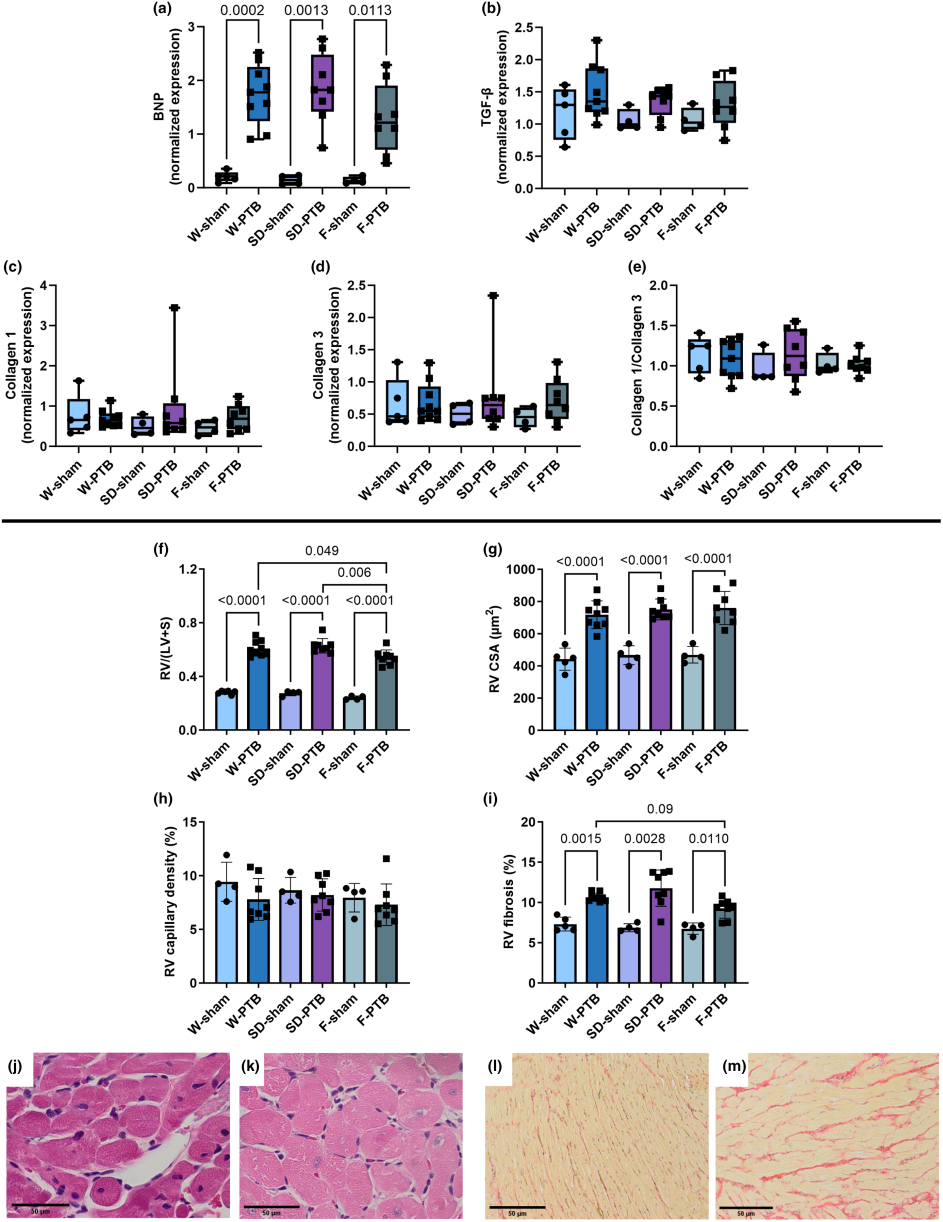
Effects of pulmonary trunk banding on heart failure markers, RV fibrosis, and RV histology. W: Wistar; SD: Sprague Dawley; F: Fischer344; PTB: Pulmonary trunk banding. (a) mRNA expression of brain natriuretic peptide (BNP). (b) mRNA expression of transforming growth factor‐β (TGF‐β). (c) mRNA expression of collagen 1. (d) mRNA expression of collagen 3. (e) collagen 1/collagen3 ratio. (f) Fulton index: Right ventricular (RV) weight/(Left ventricular (LV) + septum (S) weight). (g) RV cardiomyocyte cross sectional area (CSA). (h) RV capillary density. (i) RV fibrosis. In G‐I, one W‐sham rat is missing, further one W‐sham and W‐PTB rat are missing in H. Histological sections stained with hematoxylin and eosin from (j, l) W‐sham and (k, m) W‐PTB. Black scale bar equals 50 μm. Gene expression levels quantified by real‐time polymerase chain reaction (PCR) and normalized to 18 s and HPRT. Results presented by box plots. Anatomy and histology data are expressed as scatterplots with mean ± SD.

The PTB surgery created an immediate load increase that over time progressed to RV dysfunction with RV dilatation and systolic impairment. Already one‐week after PTB surgery, RV dysfunction was evident by decreased cardiac index (CI) and TAPSE in W‐ and SD‐PTB rats compared with their sham. F‐PTB rats only had a trend to decreased CI compared with F‐sham (*p* = 0.0954) and no decrease in TAPSE (Table S2). At end‐of‐study, an equal increase was observed in RV afterload (Ea) and RV systolic pressures in all three PTB‐groups. PTB rats showed signs of RV dysfunction evident by decreased TAPSE, CI, and velocity of RV contraction (S′). Further, an equal increase in gene expression levels of the heart failure marker, bone natriuretic protein (BNP) was evident in all PTB‐rats compared with sham. More than half of the W‐ and SD‐PTB rats developed extracardiac manifestation with nutmeg liver, ascites, and/or hydrothorax while only observed in 25% of the rats in the F‐PTB group. Further, F‐PTB rats had significantly less affected TAPSE than the two other PTB groups (Table 1, Figures 2 and 3).

RV adaptation with increased contractility was evident in the PTB rats as increased dP/dt maximum and end‐systolic elastance (Ees), though the latter nonsignificant. However, the increase in contractility was not sufficient to fully maintain the ventriculo‐arterial coupling (Ees/Ea), though it only significantly decreased in SD‐PTB compared with SD‐sham. MRI strain measures indicated impaired RV global circumferential strain (GCS) and to a lesser extent RV global longitudinal strain (GLS) in PTB rats. Again, F‐PTB rats were least effected on both strain parameters compared with W‐ and SD‐PTB. On volume parameters, W‐ and SD‐PTB had increased end‐systolic volume index' (ESVI), whereas ESVI in F‐PTB rats increased to a lesser extent and F‐PTB rats had significantly lower end‐diastolic volume index (EDVI) than W‐PTB (Table 1, Figure 2).

To summarize, W‐ and SD‐PTB rats developed similar degrees of RV failure with increased RV volumes, RV systolic pressures, and impaired RV systolic function. On the other hand, F‐PTB rats were less affected on volume parameters and more able to uphold their systolic function (TAPSE and RV global circumferential strain) despite an equal increase in RV pressure.

### 3.2 Right ventricular diastolic function

**FIGURE 4:**
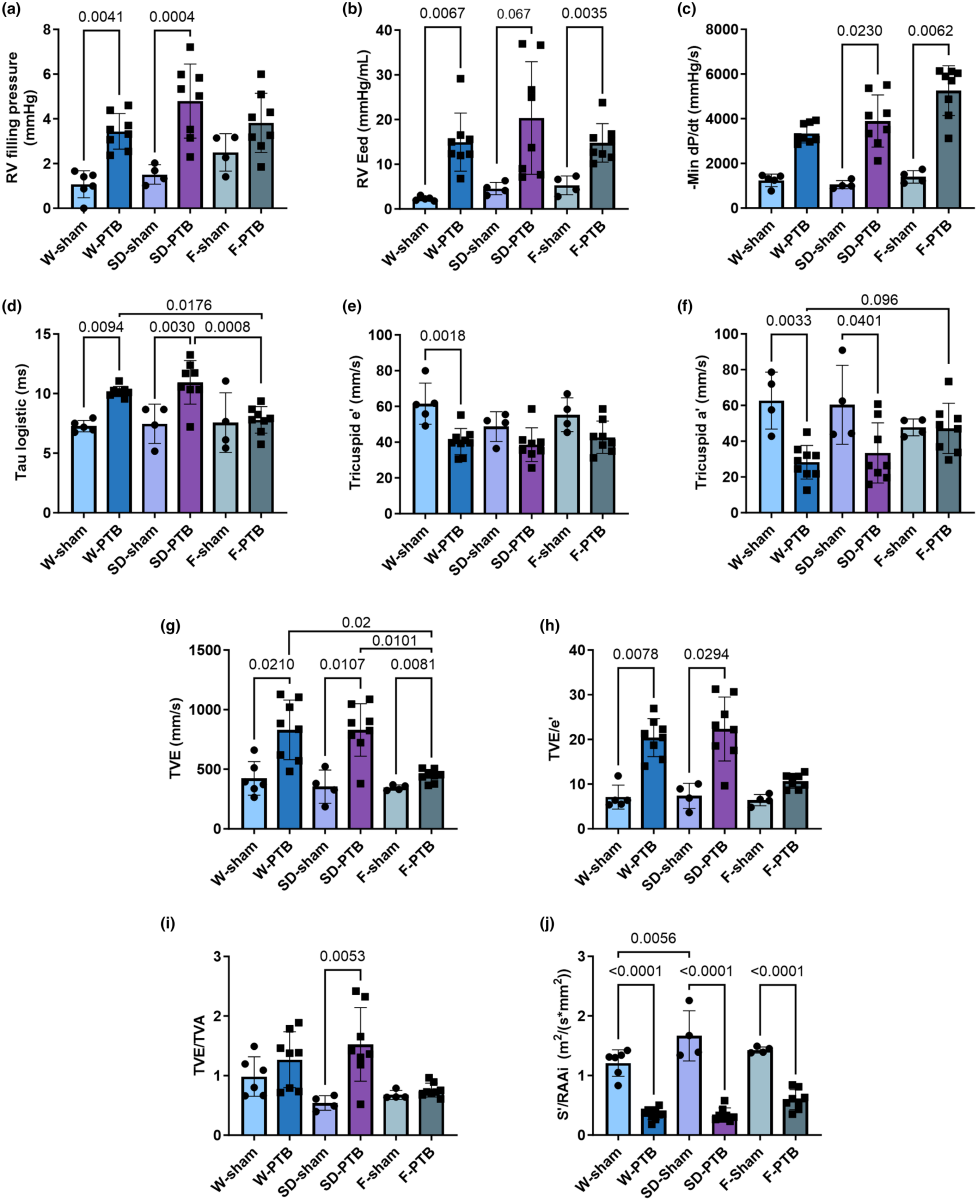
Right ventricular diastolic dysfunction is evident 5 weeks after pulmonary trunk banding surgery. W: Wistar; SD: Sprague Dawley; F: Fischer344; PTB: Pulmonary trunk banding. Hemodynamic results from pressure‐volume loops: (a) Right ventricular (RV) filling pressure as (end‐diastolic pressure)–(beginning of diastolic pressure); (b) RV end‐diastolic elastance; (c) First derivative (minimal) of RV systolic pressure (absolute values); (d) The relaxation time constant, Tau logistic. Following results derived from echocardiography: (e) Early peak of RV relaxation velocity, one rat is missing in W‐sham due to low quality; (f) Late peak of RV relaxation velocity, two rats are missing in W‐sham due to low quality; (g) Early peak of diastolic transtricuspid valve (TV) inflow; (h) RV filling pressure as TVE/e'; (i) Ratio of transtricuspid valve flow velocities. (j) Tricuspid S′/right atrial area index (RAAi). In G‐I, one W‐PTB rat missing due to EA fusion. Results are expressed as scatterplots with mean ± SD.

At end‐of‐study, PTB rats showed impaired RV filling and compliance evident by a threefold increase in RV filling pressure and increased end‐diastolic elastance (Eed) in W‐ and SD‐PTB compared with their sham, despite an adaptive increase in relaxation rate (dP/dt minimum). F‐PTB rats showed an equal increase in RV Eed and ‐dP/dt_min_. On the other hand, F‐PTB did not show a statistically significant increase in RV filling pressure and had less increased time constant of isovolumetric relaxation (Tau logistic) than the two other PTB‐groups (Figure 4).

Tissue Doppler imaging of the tricuspid lateral annulus showed decreased relaxation velocity in early (tricuspid e') and especially late diastole (tricuspid a') in W‐ and SD‐PTB compared with their sham. Early peak of transtricuspid valve (TV) inflow velocities (TVE) increased in all three PTB groups compared with their sham, albeit F‐PTB elevated to a lesser extent than the two other PTB groups. All parameters indicating increased atrial pressure and reduced RV relaxation. The noninvasive measure of RV filling pressure (TVE/e') was increased in W‐ and SD‐PTB compared with sham, though no difference between F‐PTB and F‐sham. Tricuspid S′/RAAi ratio has recently shown correlation with RV diastolic stiffness and prognostic relevance in patient with PH (Yogeswaran et al., 2023). All three PTB groups showed a severely decreased S′/RAAi ratio. SD‐PTB rats had signs of restrictive filling compared with SD‐sham indicated by increased E/A ratio (Schnelle et al., 2018). This, together with high RV filling pressures and proneness to extra cardiac manifestations (nut meg liver and fluid retention), imply a worse RV diastolic dysfunction in SD‐PTB rats (Table 1, Figure 4).

PTB caused hypertrophy evident by increased Fulton index (RV/(LV + S)) and cross sectional area (CSA) in all PTB groups compared with their sham, though F‐PTB rats had a significantly smaller increase in Fulton index. No differences in RV capillary density were observed. All three PTB groups had increased RV fibrosis compared with sham though a trend toward smaller increase in F‐PTB than in W‐PTB. However, PCR analyses did not show any differences in mRNA expression levels of genes related to collagen production for example, collagen 1, collagen 3, and TGF‐ β (Figure 3).

To summarize, one‐week after PTB surgery, only minor signs of diastolic dysfunction were evident, but at end‐of‐study W‐ and SD‐PTB showed RV filling dysfunction and increased stiffness of the heart compared with their sham. F‐PTB rats showed signs of diastolic impairment in some parameters but to a lesser extent than the other two PTB groups, indicating F‐PTB rats are more able to uphold diastolic function (Table S2, Figure 4).

### 3.3 Right atrial function

**FIGURE 5:**
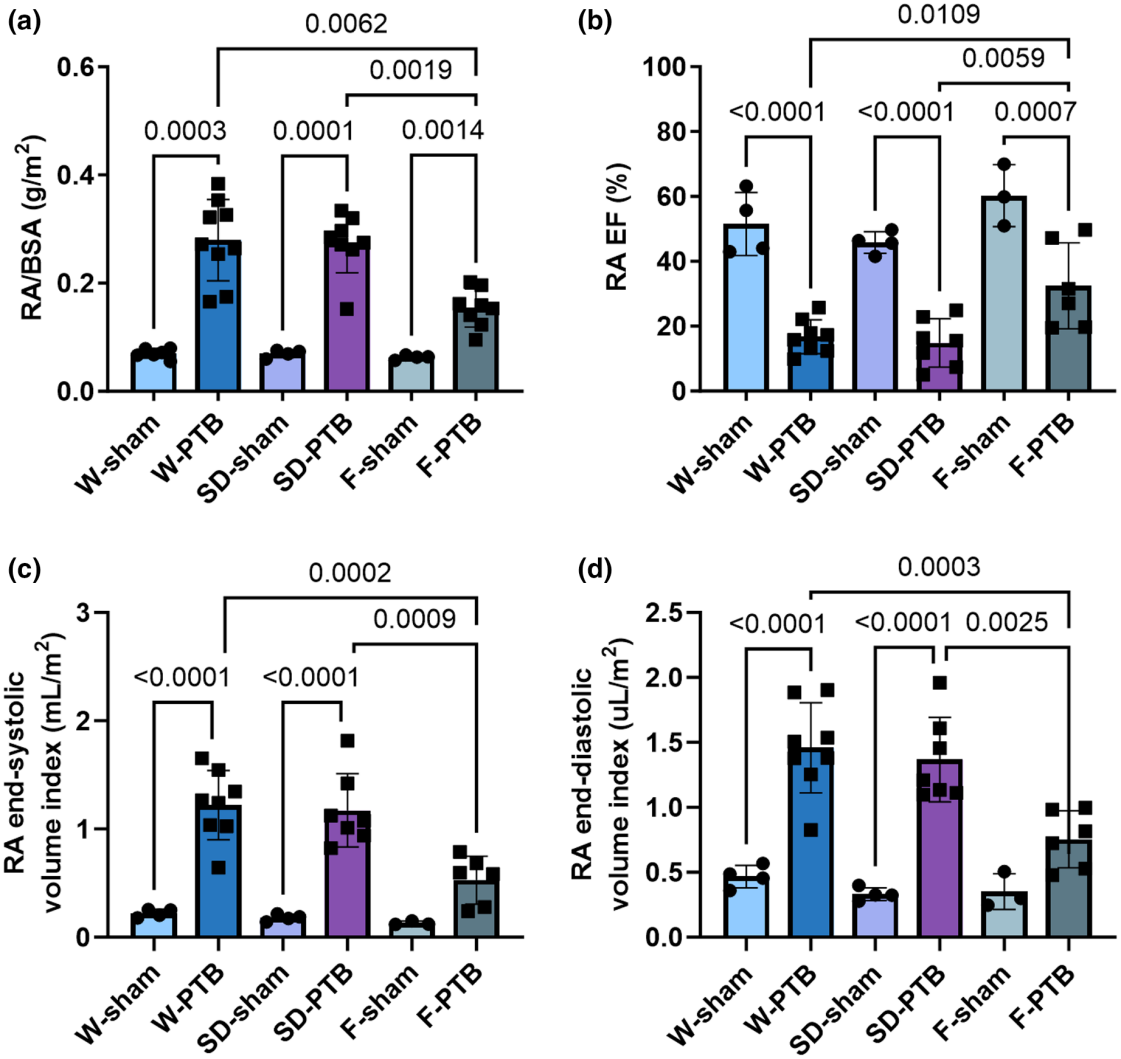
Effects of pulmonary trunk banding on RA function at end‐of‐study. W: Wistar; SD: Sprague Dawley; F: Fischer344; PTB: Pulmonary trunk banding. (a) Right atrial (RA) weight/body surface area (BSA). (b) RA ejection fraction. (c) RA end‐systolic volume index (index = volume/BSA). (d) RA end‐diastolic volume index. Due to low scanning quality one W‐sham, F‐sham, and F‐PTB are missing in RA volume index' and RA‐EF. Results are expressed as scatterplots with mean ± SD.

All PTB rats had increased size‐adjusted RA weight and hypertrophy evident by increased RA CSA compared with sham rats. W‐ and SD‐PTB caused a threefold increase in RA volume index and a reduction in ejection fraction (EF) from 50% to 20% with no differences in RA stroke volume index. When comparing with the two other PTB groups, F‐PTB rats were significantly less affected in terms of size‐adjusted RA weight, RA volume index, and a lesser RA EF reduction from 50% in F‐sham to 30% in F‐PTB. This correlates well with F‐PTB rats being able to uphold the velocity of atrial contraction (a') compared with F‐sham (Figure 4). In summary, the PTB rats showed signs of increased RA pressures, which caused increased volumes and reduced ejection fraction; the F‐PTB rats were less affected compared with the two other rat strains (Table 1, Figure 5).

## 4 DISCUSSION

We investigated rat strain differences in RV response to pressure overload and found that:

1. PTB caused RV failure evident by reduced RV function and adverse remodeling in all three rat strains.
2. Wistar and Sprague–Dawley PTB rats showed equivalent RV adaptation with both impaired systolic and diastolic function.
3. Fischer344 PTB rats showed less RV dilatation and were more able to sustain diastolic and systolic function despite similar increase in RV pressure and decrease in CI.

### 4.1 PTB is a valid model of RV failure

The PTB surgery caused increased RV afterload and RVSP in all PTB rats compared with their sham. The PTB rats showed signs of RV failure with decreased RV CI and EF caused by reduced longitudinal (TAPSE) and circumferential shortening of the RV (RV GCS). This correlates well with pressure overload induced RV failure development seen in patients, for example in PH, left ventricular failure, and congenital heart diseases (Borgdorff et al., 2015; Haddad et al., 2008). RV strain derived from cardiac MRI has shown to be a promising indicator of ventriculo‐arterial uncoupling and diastolic stiffness in patients with PH (Tello et al., 2019). Some W‐ and SD‐PTB rats developed extracardiac manifestation matching patients with RV failure, where fluid retention is one of the cardinal symptoms (Borgdorff et al., 2015; Vonk‐Noordegraaf et al., 2013). Altogether, these results validate the use of the PTB model to investigate the development of RV failure and to test new RV failure treatment strategies.

### 4.2 Rat strain differences in RV adaptation

Unexpectedly, F‐PTB rats were less affected on RV volumes and more able to sustain RV systolic and diastolic function compared with W‐ and SD‐PTB rats despite similar changes in RV pressure, CI, and mRNA expression of BNP. Histological analyses of fibrosis showed a trend toward less fibrosis in F‐PTB rats compared with W‐PTB, though this was not supported by mRNA expression levels related to collagen production. F‐sham rats already showed a trend toward differences in for example, RV volumes, TAPSE, and RV filling pressure compared with the other two strains, and we cannot exclude that these observed differences may be enhanced by the PTB surgery. Previous studies in the SuHx model of PH have shown highly increased mortality in Fischer344 rats compared with both Wistar and Sprague Dawley rats (Jiang et al., 2016; Lee et al., 2020). Shults et al, showed that Fischer344 rats developed pulmonary vascular and RV remodeling faster, but the degree of RV pressure elevation and extent of RV fibrosis were similar to Sprague Dawley rats. Fischer344 rats seem to lack the capability of adapting and repairing RV damage and show signs of inefficient cardiac energetics (Lee et al., 2020; Zelt et al., 2022). Genetic factors have been emphasized as important contributors to the susceptibility of PH (Shults et al., 2019; Suen et al., 2019). In the MCT model, on the other hand, Fischer344 rats showed less pulmonary vascular remodeling and no increase in RV pressure or RV hypertrophy (Pan et al., 1993). Others have used Fischer344 rats in the PTB model where RV end‐systolic pressure only increased to 41 mmHg with preserved cardiac output (Connelly et al., 2022). A much looser banding where used, so direct comparison with present study is difficult.

In a PH animal model, Bochnowicz et al found Wistar rats to be more susceptible to hypoxia‐induced RV hypertrophy compared with Sprague Dawley rats. They speculate that the RV hypertrophic effect is caused by a genetic predisposition to hypoxia, as they developed similar pulmonary artery pressures (Bochnowicz et al., 2000). In the present study, we did not see any indications of Wistar rats being more susceptible to pressure overload induced RV hypertrophy compared with Sprague–Dawley rats.

Based on our data, it does not seem that Fischer344 or Wistar rats have a genetically predisposition to worse pressure‐overload induced RV maladaptation. Almost on the contrary for the Fischer344 rats. Further analyses are needed to clarify the mechanisms behind, which is beyond the scope of this study. The different PH and RV models may cause different strain on the heart resulting in different RV adaptations. In the PTB model, it seems that the different banding methods, for example, clip versus ligature (Hirata et al., 2015), different banding diameters (Borgdorff et al., 2015), and the age of the rats (Kuroha et al., 1991) rather than the rat strain create the observed differences in pressure and cardiac function.

### 4.3 PTB induces diastolic dysfunction

One week after PTB surgery, the PTB rats only showed a trend toward RV diastolic dysfunction on echocardiographic parameters, though reduced TAPSE implied the onset of systolic deterioration. At end‐of‐study, PTB rats presented with RV diastolic dysfunction evident by impaired RV filling and increased RV stiffness in accordance with findings from a previous PTB study (Cheng et al., 2018). On the other hand, recent studies have shown RV diastolic dysfunction precede contractility changes in patients with PAH and in the SuHx animal model of PH (Inampudi et al., 2020; Kwan et al., 2021). Further, increased RV stiffness is correlated with several parameters depicting disease severity and can be used as marker of poor prognosis (Rain et al., 2013). A recent review by Inampudi et al. states that diastolic impairment predominantly depicts end‐stage systolic function with increased RV end‐diastolic pressure and dilated RV (Inampudi et al., 2020). Rain et al. further suggest diastolic stiffness not only to be a compensatory mechanism to increased afterload but actually contributes to disease progression affirming the need to further clarify the underlying mechanisms (Rain et al., 2013).

Currently, no consensus exists on how to fully describe RV diastolic function—especially in animal research. Ogilvie et al. demonstrate that not one diastolic parameter was sufficient to describe diastolic function and the incongruence of diastolic parameters over time could be caused by sensitivity varieties of each measure (Ogilvie et al., 2020). The noninvasive measurements of diastolic function from echocardiography and MRI are highly load‐dependent (Rain et al., 2013). So, the load‐independent gold standard of measuring diastolic stiffness is the end‐diastolic elastance (Eed). In the present study, Eed had a moderate correlation with following diastolic measures: RV filling pressure, tricuspid e', TVE/tricuspid e', dp/dt min, Tau Logistic, and tricuspid S/RAAi. A less positive correlation was seen with tricuspid a', TVE, and RV fibrosis. No correlation was seen with E/A (Figure S1). From our experience as well as others (Boehm et al., 2017), Eed can be challenging to measure especially in smaller and healthier rodents. Therefore, when assessing diastolic function, we often must assess all the different diastolic parameters on filling and active relaxation to get the full picture (Ogilvie et al., 2020). Despite F‐PTB rats had similar increase in Eed, when taken together all the other measured diastolic parameters as well as the less affected RA, it may suggest that the F‐PTB rats are better to uphold diastolic function compared with the other two rat strains.

In brief, further studies are needed to better understand the underlying mechanisms in RV diastolic dysfunction development in pressure overload induced RV failure. We show that the PTB model can be used to investigate development of RV diastolic dysfunction and test new treatment options addressing this problem.

### 4.4 PTB induces RA impairment

W‐ and SD‐PTB rats showed comparable impaired RA function with increased backward venous flow followed by liver congestion. F‐PTB rats, on the other hand, showed impaired RA function but to a lesser extent than the two other PTB groups. A recent study showed an association between severe RV diastolic stiffness and atrio‐ventricular uncoupling, RA stiffness, and hypertrophy in patients with PH (Wessels et al., 2023). Further, elevated RA pressures and enlarged RA size are risk factors for severe outcomes or death in PH, and RA area measured from a 4‐chamber view is currently in the guidelines for risk stratification in PH (Cogswell et al., 2014; Lang et al., 2022; Liu et al., 2020; Raymond et al., 2002). RA impairment seems to be caused by RV diastolic dysfunction rather than RV contractility, but the causality is still unclear (Richter et al., 2020; Tello et al., 2019).

## 5 STRENGTHS AND LIMITATIONS

This study has several strengths: (1) We used a well‐established model of isolated RV failure using a ligating clip to constrict the PT (Andersen et al., 2018). (2) A wide variety of clinically relevant modalities such as echocardiography, cardiac MRI, and pressure‐volume measurements were used to evaluate hemodynamic profiles.

All three rat strains (only males) were purchased from Janvier Labs to exclusively evaluate rat strain differences. The same rat strains but from other animal suppliers may differ (Lassen et al., 2021). Rat strain differences in RV adaptation to pressure overload in female rats needs to be assessed in future studies. Sex differences to the PTB surgery have previously been investigated in Wistar rats (Labazi et al., 2020). The use of inbred rat strains, such as Fischer344 rats, has the potential to minimize inter‐animal variation making the genotype more predictable so fewer animals are needed in experiments. Though, they have proved more susceptible to hereditary abnormalities and reduced fertility and weight (Festing, 2014). Data from present study do not show any differences in rat strain variance throughout the different measurements implying that outbred rat strains are suitable to use in the PTB model. The main focus of present study was to explore differences in RV failure development after PTB, so a relative low number of sham rats were included to minimize the number of rats used according to the principle of the three R's. However, the limited number of sham rats in each group may introduce bias and reduce strengths when comparing the sham groups. All hemodynamic measures were conducted in anesthetized animals, and to minimize the anesthetic effects on our results a thoroughly tested protocol was followed in all animals.

## 6 CONCLUSION

PTB caused RV failure in all rats subjected to the procedure including signs of diastolic dysfunction. W‐ and SD‐PTB developed equivalent RV dysfunction validating comparisons of RV adaptation between these two rat strains. A reassuring finding in PH animal research as these are the most frequently used rat strains. Despite similar increase in RV systolic pressure, F‐PTB rats showed less RV dilatation and were more able to sustain systolic and diastolic function. All three rat strains are suitable for PTB and studies of RV failure. The present study accentuates the importance of careful consideration when selecting rat strain during experiments and comparing papers to pave the way for improved quality and transferability in animal research. Here, the use of several animal models and different rat strains may aid the translatability.

## AUTHOR CONTRIBUTIONS

JSA, SA, FSdM, AA conceived and designed research; JSA, RS, ALV, and SR performed experiments; JSA analyzed data; JSA, SA, JENK, AA interpreted results of experiments; JSA prepared figures; JSA drafted manuscript; SA, FsDM, JENK, RS, ALV, SR, and AA edited and revised manuscript; SA, FsDM, JENK, RS, ALV, SR, and AA approved final version of manuscript.

## FUNDING INFORMATION

JSA have received funding from Hjerteforeningen (Heart Foundation) 20‐R140‐A9671‐22167; The Danish Medical Research Grant 2017–1064/96; A.P. Moeller Fonden 20‐L‐0184; Helga og Peter Kornings Fond (Helga and Peter Korning's Fund) DC472123‐004‐lek; Soester og Verner Lipperts Fond 25/11–2021; Snedkermester Sophus Jacobsen og hustru Astrid Jacobsens Fond J167/1. FSdM was supported by the Netherlands CardioVascular Research Initiative: the Dutch heart foundation, Dutch federation of university medical centres, the Netherlands organization for health research and development, and the royal Netherlands academy of sciences (CVON‐2018‐29 phaedra‐impact and CVON‐2017‐10 dolphin‐genesis). FSdM was further supported by the Netherlands organization for scientific research (NWO‐VIDI: 917.18.338) and the Dutch heart foundation dekker senior postdoc grant (2018 t059).

## CONFLICT OF INTEREST STATEMENT

Prof.dr de Man has received research grant support from Janssen and BIAL. Asger Andersen has received research grant support from BIAL and teaching honorariums from EPS vascular, ABBOTT, Angiodynamics, Inari Medical, Gore Medical, and Janssen. All other authors have declared that they have no conflict of interest, financial, or otherwise.

## ETHICS STATEMENT

All animal experiments were approved by the Institutional Ethics Review Board, the Danish Animal Experiments Inspectorate, and conducted in accordance with the Danish law for animal research (authorization number 2021‐15‐0201‐00928, Ministry of Environment and Food of Denmark).

## Supporting information


Data S1.

## Data Availability

The data that support the findings of this study are available from the corresponding author upon reasonable request.
