# Supplementary material for: Right ventricular diastolic adaptation to pressure overload in different rat strains
Source: Physiol Rep. 2024 Jul 11;12(13):e16132. doi: 10.14814/phy2.16132 (PMC11239975; doi:10.14814/phy2.16132)
Supplement: Supplementary file 1 — Data S1. [file PHY2-12-e16132-s001.docx]

**Supplementary tables and figures**

| **Primer sequences** | **Forward** | **Reverse** |
| --- | --- | --- |
| HPRT | CTCCTCAGACCGCTTTTCCC | TAATCACGACGCTGGGACTG |
| 18s | CTCTTCCACAGGAGGCCTACAC | TGAGTACTCGCAGGATGTGC |
| Collagen 1A1 | GAACGGAGATGATGGGGAAG | CCAAACCACTGAAACCTCTG |
| Collagen 3a | AGTGGCCATAATGGGGAACG | CAGGGTTTCCATCCCTTCCG |
| BNP | GCTGCTTTGGGCAAGAAGATAGA | GCCAGGAGGTCTTCCTAAAACA |
| TGF-β | ATGCCAACTTCTGTCTGGGG | CCCGGGTTGTGTTGGTTGTA |

**Supplemental Table S1: Primer sequences for real-time polymerase chain reaction**. HPRT: hypoxanthine guanine phosphoribosyltransferase; BNP: brain natriuretic peptide; TGF-β: transforming growth factor-β.

|  | **W-sham**  n = 6 | **W-PTB**  n = 9 | **SD-sham**  n = 4 | **SD-PTB**  n = 8 | **F-sham**  n = 4 | **F-PTB**  n = 9 |
| --- | --- | --- | --- | --- | --- | --- |
| BW week 1 (g) | 196 ± 9 | 180 ± 21 | 196 ± 62 | 186 ± 19 | 142 ± 11 | 137 ± 18 |
| HR (bmp) | 346 ± 40 | 304 ± 25 | 344 ± 36 | 293 ±18* | 343 ± 19 | 287 ± 25* |
| CI (mL/min/m^2^) | 6.0 ± 0.6 | 3.3 ± 0.4**** | 6.6 ± 1.1 | 3.2 ± 0.3* | 5.9 ± 1.1 | 3.7 ± 0.9 |
| Pulm dia (mm) | 2.81 ± 0.15 | 2.83 ± 0.15 | 2.79 ± 0.14 | 2.83 ± 0.16 | 2.69 ± 0.06 | 2.78 ± 0.25 |
| TAPSE (mm) | 2.5 ± 0.3 | 1.8 ± 0.3**** | 2.4 ± 0.3 | 1.7 ± 0.3** | 2.2 ± 0,1 | 2.0 ± 0.2 |
| RA (mm^2^) | 21 [19-23] | 35 [29-42] | 21 [18-26] | 36 [34-41] | 15 [13-17] | 21 [19-27] |
| E/e’ | 7.6 ± 3.8 | 11.8 ± 3.7† | 8.9 ± 0.1 | 11.5 ± 2.6† | 5.8 ± 1.5 | 7.1 ± 1.9 |
| TR | 0 (0) | 7 (78)** | 0 (0) | 6 (75) | 0 (0) | 3 (38) |

**Supplemental Table S2: Echocardiography data from one week after pulmonary trunk banding surgery.** W: Wistar; SD: Sprague Dawley; F: Fischer344; PTB: pulmonary trunk banding; BW: body weight; HR: heart rate; CO: cardiac output; Pulm dia: pulmonary diameter; TAPSE: Tricuspid annular plane systolic excursion; RA: right atrium; E/e’: tricuspid E/e’ ratio; TR: tricuspid regurgitation. Missing data in E/e’ from two W-sham, one W-PTB, two SD-sham rats due to EA fusion.

Normally distributed data are presented as mean ± standard deviation (SD) and non-normally distributed data as median [interquartile range]. Dichotomous data are presented as *n* (%). * p < 0.05; ** p < 0.01; **** p < 0.0001 PTB vs sham within same strain. ^ p < 0.05; ^^ p < 0.01 sham vs F-sham. † p < 0.05; †† p < 0.01 PTB vs F-PTB.

|  | **W-sham**  n = 5 | **W-PTB**  n = 8 | **SD-sham**  n = 4 | **SD-PTB**  n = 8 | **F-sham**  n = 4 | **F-PTB**  n = 8 |
| --- | --- | --- | --- | --- | --- | --- |
| Lungs/BSA (g/m^2^) | 2.3 [2.1-2.7] | 2.5 [2.2-2.6] | 2.4 [2.3-2.5] | 2.6 [2.2-2.6] | 2.1 [2.1-2.3] | 2.3 [2.2-2.9] |
| Kidneys/BSA (g/m^2^) | 4.9 ± 0.19 | 4.3 ± 0.30*** | 5.0 ± 0.24^^^ | 4.3 ± 0.24*** | 4.1 ± 0.19 | 4.1 ± 0.17 |
| Spleen/BSA (g/m^2^) | 1.9 [1.5-2.4] | 1.8 [1.5-2.0] | 1.8 [1.6-1.9] | 2.2 [1.8-2.8]† | 1.3 [1.3-1.8] | 1.6 [1.4-1.7] |
| Pulm dia (mm) | 3.1 [3.1-3.2] | 2.9 [2.8-3.0]* | 3.2 [3.1-3.3] | 3.0 [2.9-3.1] | 3.1 [3.0-3.2] | 3.0 [2.9-3.1] |

**Supplemental Table S3: Data at end-of-study.** W: Wistar; SD: Sprague Dawley; F: Fischer344; PTB: pulmonary trunk banding; BSA: body surface area; Pulm dia: pulmonary diameter, measured proximally before the narrowing of the clip.

Normally distributed data are presented as mean ± standard deviation (SD) and non-normally distributed data as median [interquartile range]. * p < 0.05; *** p < 0.001 PTB vs sham within same strain. ^^^ p < 0.001 sham vs F-sham. † p < 0.05 PTB vs F-PTB.


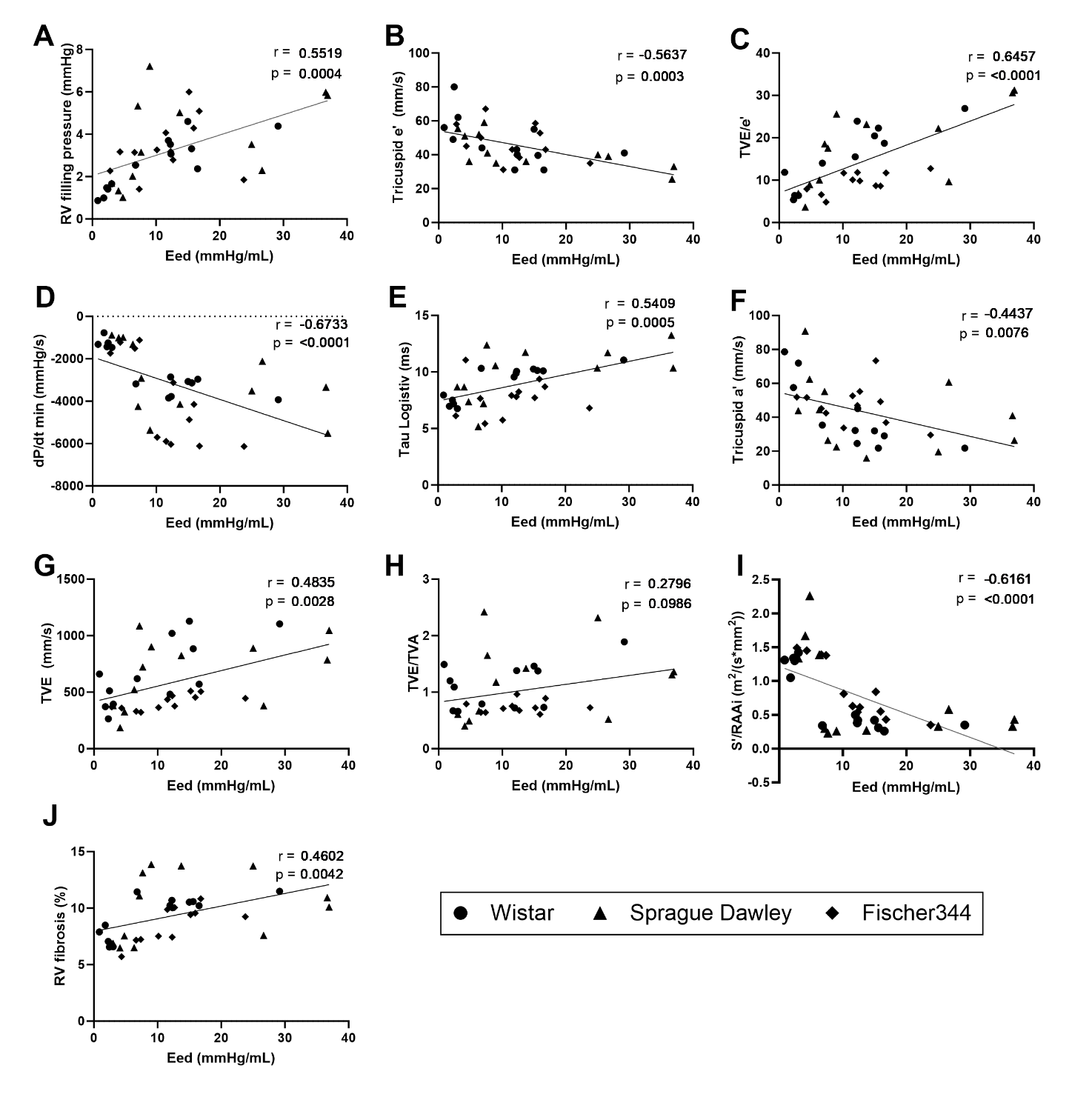
**Supplemental Fig S1: End-diastolic elastance (Eed) correlations.** A) Correlation between Eed and RV filling pressure. B) Correlation between Eed and tricuspid e’. C) Correlation between Eed and tricuspid valve E (TVE)/e’. D) Correlation between Eed and dP/dt minimum. E) Correlation between Eed and Tau Logistic. F) Correlation between Eed and tricuspid a’. G) Correlation between Eed and TVE. H) Correlation between Eed and TVE/TVA. I) Correlation between Eed and tricuspid S’/(right atrial area index (RAAi)). J) Correlation between Eed and RV fibrosis.
